# Predictors of motor complications in early Parkinson's disease: A prospective cohort study

**DOI:** 10.1002/mds.27783

**Published:** 2019-07-08

**Authors:** Mark J. Kelly, Michael A. Lawton, Fahd Baig, Claudio Ruffmann, Thomas R. Barber, Christine Lo, Johannes C. Klein, Yoav Ben‐Shlomo, Michele T. Hu

**Affiliations:** ^1^ Oxford Parkinson's Disease Centre University of Oxford Oxford UK; ^2^ Nuffield Department of Clinical Neurosciences University of Oxford Oxford UK; ^3^ Population Health Sciences University of Bristol Bristol UK; ^4^ Neurology Department Hampshire Hospitals National Health Service (NHS) Foundation Trust Basingstoke UK

**Keywords:** dyskinesia, levodopa, motor complications, motor fluctuations, Parkinson's disease

## Abstract

**Objective:**

The objective of this study was to identify clinical predictors of motor complications (dyskinesia and motor fluctuations) of levodopa in a prospectively recruited PD cohort using longitudinal analysis.

**Methods:**

An inception cohort (Oxford Discovery) of 734 patients was followed to a maximum of 10 years from diagnosis using a discrete‐time survival analysis. A subset analysis was used to validate an online dyskinesia‐risk calculator developed from the results of the Stalevo Reduction in Dyskinesia Evaluation PD trial.

**Results:**

A total of 186 cases of dyskinesia and 254 cases of motor fluctuations were observed. Dyskinesia incidence increased with time (risk per 100 participants [95% confidence interval] 13 [11–16] <3.5 years, 16 [13–21] 3.5–5.0 years, 19 [14–26] 5–6.5 years, and 23 [16–33] >6.5 years from diagnosis). Motor complication predictors were grouped as medication predictors, disease predictors and patient predictors. Baseline nonmotor feature severity, low mood, anxiety, and age at symptom onset were associated with motor complications among a number of previously identified predictors. Replication of the Stalevo Reduction in Dyskinesia Evaluation PD calculator was reasonable with the area under the curve for dyskinesia risk score as a predictor of dyskinesia being 0.68 (95% confidence interval, 0.55–0.81).

**Conclusions:**

This study quantifies risk of motor complications, finds consistent predictors, and demonstrates the novel finding that nonmotor features of PD, particularly low mood and anxiety, are significant risk factors for motor complications. Further validation of dyskinesia risk scores are required as well as evidence to determine if the routine use of such scores can be clinically valuable in enhancing patient care and quality of life. © 2019 The Authors. *Movement Disorders* published by Wiley Periodicals, Inc. on behalf of International Parkinson and Movement Disorder Society.

Levodopa, the gold‐standard symptomatic treatment in Parkinson's Disease (PD), can have significant motor complications, namely, dyskinesia and motor fluctuations (MF).^1^ The vast majority of patients experience motor complications by 15 years of treatment, although they can occur within a year of commencement,^2^, ^3^ impacting quality of life and influencing treatment decisions. Levodopa treatment is often delayed and replaced by less effective, poorly tolerated alternatives, such as dopamine agonists (DA).^4^ It is therefore important to identify those at greater risk of motor complications. Attempts have been made to develop a dyskinesia risk calculator^5^ that can be used in clinical practice.

The evidence on predictors of motor complications is variable, often contradictory, and typically from cross‐sectional studies^6^ or secondary outcomes in clinical trials^7^ of late‐stage PD.^2^ There are few longitudinal cohort studies,^8^, ^9^ and even fewer are prospectively recruited.^10^, ^11^

Our aim was to identify risk factors for the onset of motor complications in the Oxford Parkinson's Disease Centre Discovery cohort, a large, prospectively recruited, clinic‐based cohort of early PD participants, using a comprehensive and robust longitudinal survival analysis model.

## Materials and Methods

### Study Population

Details of recruitment to the Discovery cohort have been described elsewhere.^12^ PD patients meeting the UK Brain Bank criteria for diagnosis^13^ (but not other forms of nonidiopathic parkinsonism) were recruited from neurology clinics across Thames Valley in the United Kingdom between September 2010 and September 2014 within 3.5 years of diagnosis, regardless of age or family history. Participants who developed dementia within 1 year of diagnosis were excluded as having dementia with Lewy bodies. Cases were included if they had a diagnostic certainty of PD greater than 90% at their most recent visit, were taking levodopa, and had data available past their first year after diagnosis.

The study was undertaken with the understanding and written consent of participants, approval of the local National Health Service (NHS) research ethics committee, and in compliance with national legislation and the Declaration of Helsinki.

### Outcomes

Data were collected prospectively using patient‐completed and interviewer‐completed questionnaires and clinical examination every 18 months.

The presence and intensity of motor complications was determined using the Movement Disorder Society–Sponsored Revision of the Unified Parkinson's Disease Rating Scale (MDS‐UPDRS) IV^14^ questions 1 (dyskinesia) and 3 (MF). Dyskinesia was also recorded if witnessed by the interviewer. Functional impact of complications was determined using questions 2 and 4. Participants were considered to have both dyskinesia and MF from the time of onset of the second motor complication. Missing data were excluded from the analysis.

### Other Covariates

A range of covariates were selected for regression modelling based on a priori hypothesis. Details of the collection methods for these covariates are described in Supporting Information Table S1.

Because PD carries a significant nonmotor symptom burden, a range of nonmotor features and their severity, as measured by the MDS‐UPDRS I, were also tested. These features (cognition, anxiety, depression, sleep disturbance, constipation, hyposmia, autonomic disturbance, and impulsivity) can occur as a consequence of PD, its treatments, or both and provide insight into an individual's parkinsonian phenotype. Patient factors (age, gender, body mass index [BMI], smoking status, caffeine use, and socioeconomic status) also influence this phenotype and were hypothesized to influence motor complication risk.

### Statistical Analysis

Data were extracted (May 11, 2017), checked, and cleaned.

A discrete‐time survival analysis (DTSA) model was used rather than more conventional continuous‐time methods because the exact date of motor complication onset is liable to recall bias when collecting data retrospectively. This model uses censoring based on the date of clinical assessment. Four clinically relevant intervals based on time since PD diagnosis— < 3.5 years, 3.5 to 5 years, 5 to 6.5 years, and ≥ 6.5 years (<10 years)—were selected. Data for each case were only included once per time interval; cases were not required to have data for every interval. If participants attended twice in an interval, the data were taken from their most recent visit or when motor complications were first recorded. Cases were censored at the last visit or when motor complications were recorded. Hazard risk and estimated conditional survival risks were calculated for motor complications at each time interval. The latter is the risk −1 for the observed time period multiplied by the survival risk in the previous time period, hence the conditional probability. Cases were only entered into time intervals during which the participant was taking levodopa.

Logistic regression analyses used onset of motor complication as a dichotomous dependent variable and time as an independent categorical variable, predicting the hazard rate of motor complications over time. This is the DTSA equivalent of a Cox regression analysis. Univariable exploratory analyses were performed using the covariates described. Age at diagnosis, age at symptom onset, gender, and smoking history were included as time‐fixed variables, so their value for a single case was unchanged across all time intervals. All other covariates were time varying, taking on the appropriate value for each case at each time interval. Additional analyses using baseline, time‐fixed measures of anxiety, depression, and MDS‐UPDRS I were performed to test the directionality of effect of these variables. In these analyses, outcome data were taken only from follow‐up, not baseline visits.

Multivariable stepwise regression analyses with backward elimination were performed using independent variables selected on the basis of a priori hypotheses and results from univariable analyses. The DTSA model assumes that the effect of any independent variable is proportional overtime. For significant (*P* < 0.05) variables on univariable analysis, this assumption was tested by comparing goodness of fit between models using a completely general (by calculating four different hazard risk values for a variable, 1 for each discrete time interval) or linear association with time (by including the interaction term “variable × time‐1,” where “time” is an ordinal variable representing the 4 discrete time intervals). If an association improved the model significantly (*P* < 0.05, likelihood ratio test), it was included in the multivariable model.

A subset analysis was designed to validate the dyskinesia risk calculator proposed by Schapira and colleagues.^5^ based on the results from the Stalevo Reduction in Dyskinesia Evaluation–PD (STRIDE‐PD) trial.^7^ The included cases met the same characteristics as those in that study, that is, levodopa‐naive at the start of the study and subsequently started on levodopa, no treatment changes in the 4 weeks preceding levodopa initiation, no recent amantadine, and naive to catechol‐O‐methyltransferase inhibitors before levodopa initiation. The independent variables were gender, age (at levodopa commencement), levodopa dose (at first visit after levodopa commencement), weight (at first visit after levodopa commencement), and MDS‐UPDRS II (prior to levodopa commencement). The formula for calculating dyskinesia risk is the following:

$$\begin{array}{l} (–0.04724\times \mathrm{age}\;\text{in years}\;\\ +0.10965\times \text{levodopa dose}\;\left[\mathrm{mg}\right]\;\mathrm{per}\;\text{weight}\;\left[\mathrm{kg}\right]\\ +0.04336\times \text{UPDRS}\;\mathrm{II}\;\text{score}\;\\ +0.38902\;\left[\text{if female}\right]+3.5)\times 27.5.\; \end{array}$$

Participants were included if they were observed between 140 and 200 weeks of treatment or if dyskinesia onset was earlier. This time interval captured cases as close as possible to the 169‐week end point used by STRIDE‐PD while achieving an adequate sample size. Sensitivity analyses were performed using intervals of 150 to 190 and 0 to 200 weeks. Receiver operating curve analysis for risk score as a predictor of dyskinesia was performed.

Analysis was performed using SPSS version 23 (IBM Corp., Armonk, NY).

## Results

A total of 947 participants were recruited to the Oxford Parkinson's Disease Centre Discovery cohort; 91 were not followed up past 1 year from the date of PD diagnosis. A total of 107 participants never took levodopa, and 9 had insufficient data for inclusion. Of the total participants, 740 were eligible for the study; 734 and 733 participants were included in the dyskinesia and MF analyses, respectively, as they had appropriate outcome data.

Of the 740 participants, 260 (35.1%) were women. Mean age at baseline was 64.89 years (range 29.70–87.45 years, SD = 9.51). Mean time since diagnosis was 1.35 years (range 0.04–3.49 years, SD = 0.96). Mean time since motor symptom onset was 3.0 years, with a small number of right‐sided outliers (range 0.2–13.9 years, interquartile range 1.8–3.8 years, SD = 1.9). Of 726 participants, 366 (49.5%) were tremor‐phenotype dominant. Distribution of Hoehn and Yahr staging was as follows: 158 participants (21.4%) stage 1, 521 (70.4%) stage 2, 59 (8.0%) stage 3. Of 732 participants 101 (13.8%) scored <22 on the Montreal Cognitive Assessment (possible dementia).

**Figure 1:**
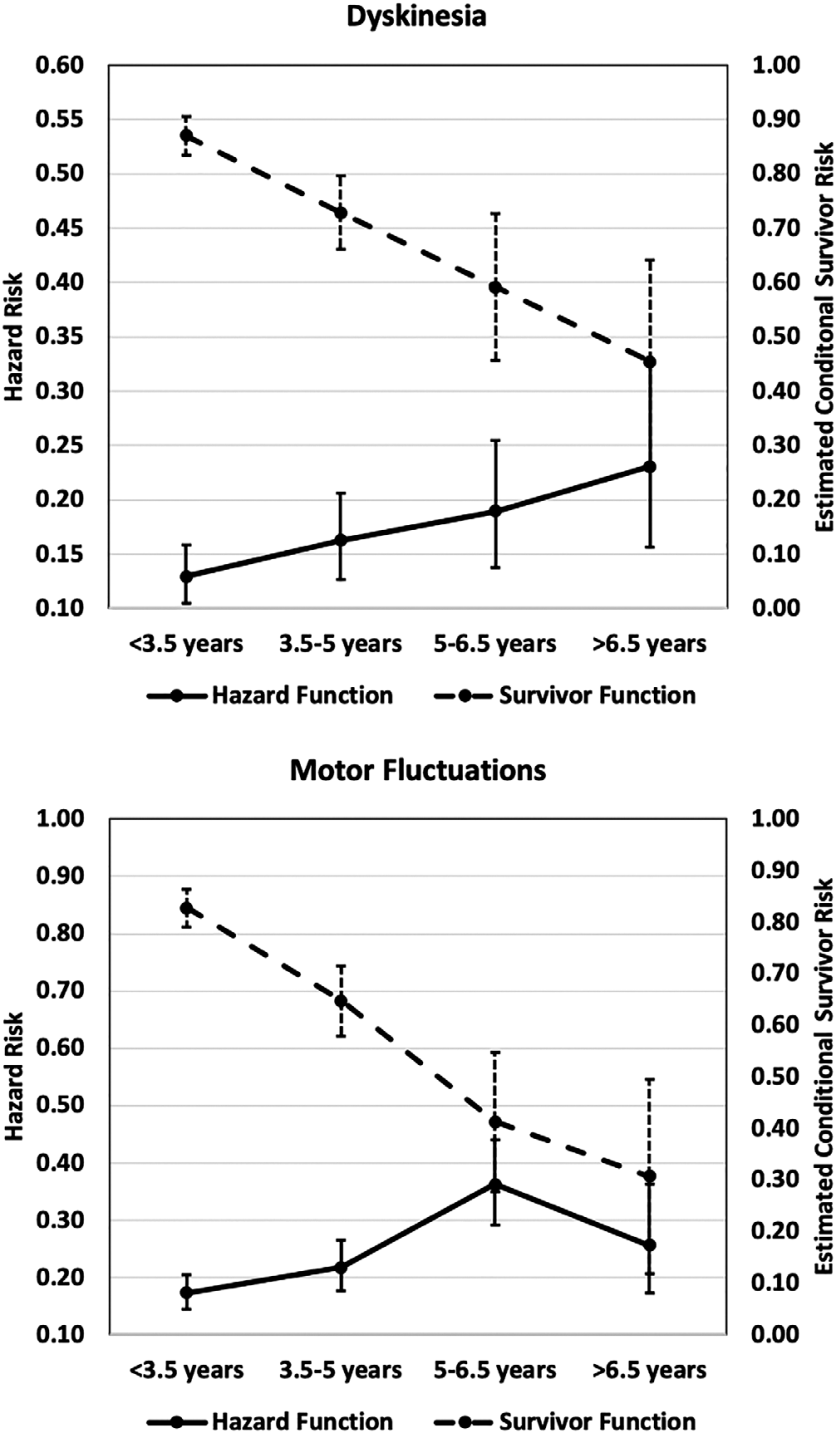
Hazard and survivor functions for dyskinesia and motor fluctuations in Parkinson's disease. Hazard functions correspond to left vertical axis and survivor functions to the right axis. Error bars; 95% confidence interval. The hazard function illustrates probability (point prevalence) of new motor complication onset within each time interval. The survivor function illustrates cumulative risk over time, in the same way as a Kaplan‐Meier curve.

**Figure 2:**
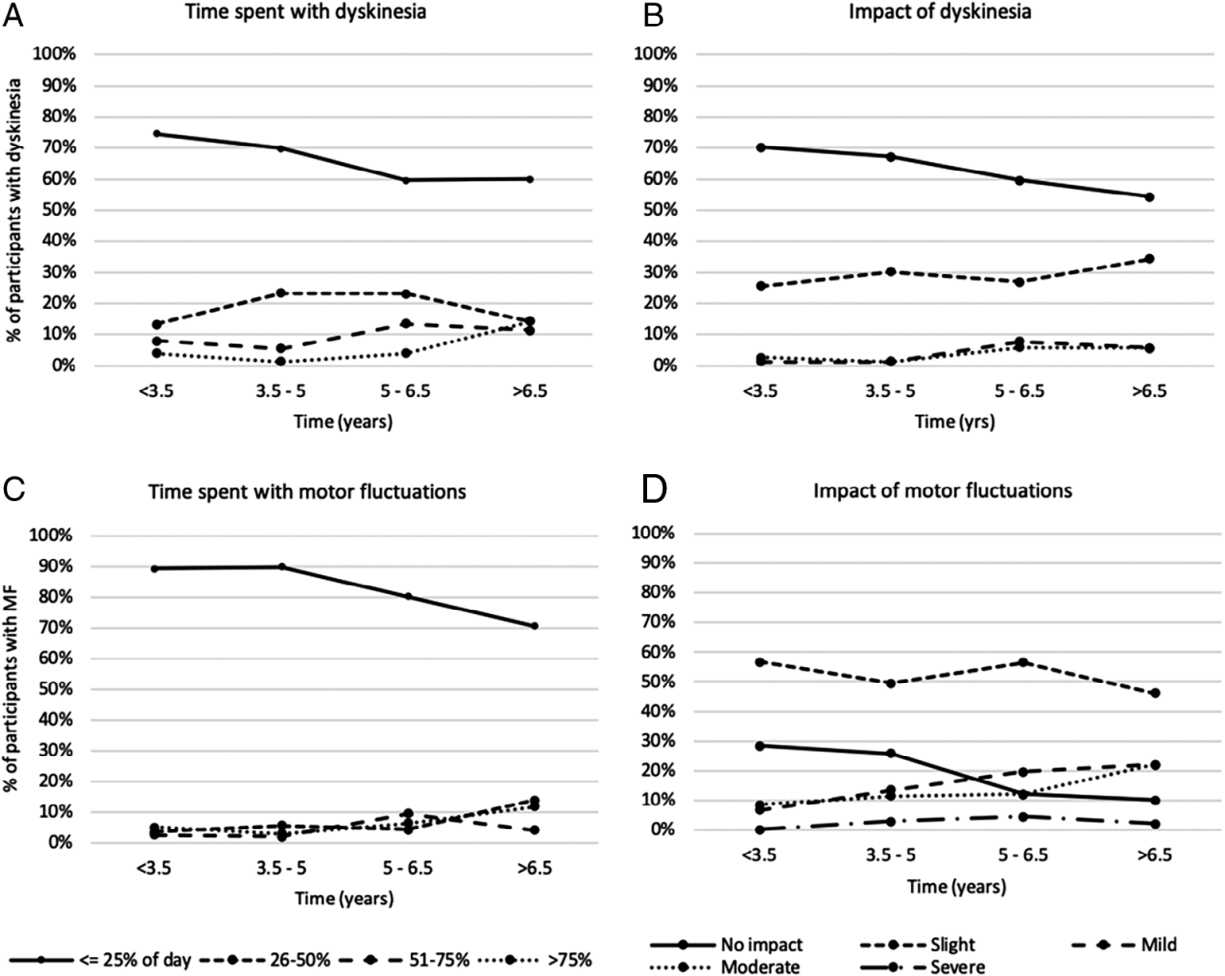
Time spent with, and functional impact of, motor complications (among participants with motor complications).

Table 1 shows the incidence of motor complications over time. A total of 186 (25.3%) of 734 cases of dyskinesia and 254 (34.7%) of 733 cases of MF were observed. Figure 1 illustrates the hazard and estimated conditional survival risks. The hazard risk represents the predicted probability of new onset motor complication at each time interval. The estimated conditional survival risk represents the probability of not developing a motor complication through each time interval and is interpreted in the same way as a Kaplan‐Meier curve (ie, cumulative risk). Figure 2 illustrates the intensity and impact of motor fluctuations.

**Table 1:** Life table for the onset of new dyskinesia and motor fluctuations over time

		Time from Diagnosis			
		<3.5 years	3.5‐5 years	5‐6.5 years	>6.5 years
	**Median time since diagnosis (years)**	2.53	4.19	5.64	7.33
**Dyskinesia**	**N on levodopa**	610	332	169	91
	**N with new dyskinesia**	79	54	32	21
	**Hazard Risk**	0.13	0.16	0.19	0.23
	**(95% CI)**	(0.11, 0.16)	(0.13, 0.21)	(0.14, 0.26)	(0.16, 0.33)
	**Conditional Survivor Risk (estimated** a)	0.87	*0.73*	*0.59*	*0.45*
	**(95% CI)**	(0.79, 0.86)	*(0.58, 0.71)*	*(0.28, 0.55)*	*(0.12, 0.49)*
	**With previous or concurrent MF (%)** **(95% CI)**	23/79 (29.1%) (20.3%, 39.0%)	36/54 (66.7%) (53.4%, 77.8%)	20/32 (62.5%) (45.3%, 77.1%)	17/21 (81.0%) (60.0%, 92.3%)
**Motor Fluctuations**	**N on levodopa**	613	322	160	78
	**N with new MF**	106	70	58	20
	**Hazard Risk**	0.17	0.22	0.36	0.26
	**(95% CI)**	(0.15, 0.20)	(0.18, 0.27)	(0.29, 0.44)	(0.17, 0.36)
	**Conditonal Survivor Risk (estimated** a)	0.83	*0.65*	*0.41*	*0.31*
	**(95% CI)**	(0.80, 0.86)	*(0.59, 0.70)*	*(0.34, 0.49)*	*(0.22, 0.42)*
	**With previous or concurrent dyskinesia (%)** **(95% CI)**	22/106 (20.8%) (14.1%, 29.4%)	28/70 (40.0%) (29.3%, 51.8%)	27/58 (46.6%) (34.3%, 59.2%)	12/20 (60.0%) (38.7%, 78.1%)
**Dyskinesia and Motor Fluctuations**	**N on levodopa**	606	337	165	85
	**N with dyskinesia and MF**	27	42	34	21
	**Hazard Risk**	0.04	0.12	0.21	0.25
	**(95% CI)**	(0.03, 0.06)	(0.09, 0.16)	(0.15, 0.27)	(0.17, 0.35)
	**Conditonal Survivor Risk (estimated** a)	0.96	*0.84*	*0.66*	*0.50*
	**(95% CI)**	(0.94, 0.97)	*(0.79, 0.88)*	*0.57, 0.75)*	*(0.35, 0.65)*

Participants were considered to have experienced dyskinesia and MF from the time at which the second motor complication was observed.

95% CI; 95% Confidence interval. Calculated for estimated survivor ratio using Greenwood formula.

a To adjust for censoring after the first time point, estimated survivor ratios are presented as a factor of the previous survivor ratio.

### Predictors of Motor Complications

Tables 2 and 3 illustrate the results of backward stepwise logistic regression analyses.

**Table 2:** Predictors of dyskinesia

	Variables	Univariable		Multivariable	
		Exp(B) (95% CI)	*P*	Exp(B) (95% CI)	*P*
**Time (Yrs)**	**Time**		0.04		0.49
	<3.5				
	3.5‐5	1.31 (0.90, 1.9)	0.16	1.12 (0.75, 1.68)	0.58
	5‐6.5	1.57 (1.00, 2.47)	0.05	1.17 (0.70, 1.95)	0.55
	≥6.5	2.02 (1.17, 3.47)	0.011	1.61 (0.88, 2.97)	0.12
**Medication Factors**	**Levodopa** **dose (mg/100)** a	1.20 (1.13, 1.28)	<0.001	1.17 (1.09, 1.25)	<0.001
	**Medication response** a (1‐Very much improved – 7‐very much worse)	0.55 (0.45, 0.66)	<0.001	0.61 (0.49, 0.75)	<0.001
	**Levodopa** **treatment** **duration (Years)** a	1.05 (0.97, 1.14)	0.24		
	**DA use** a	0.91 (0.65, 1.28)	0.60		
	**MAOBI use** a	1.24 (0.89, 1.72)	0.20		
**Motor Features**	**MDS‐UPDRS II (/5)** ^**a**^	1.02 (1.00, 1.044)	0.059		
	**MDS‐UPDRS III (/5)** ^**a**^	0.99 (0.97, 1)	0.044		
	**Disease Phenotype** a (Tremor Dominant)	0.71 (0.51, 0.99)	0.044	‐	‐
**Non‐motor Features**	**MDS‐UPDRS I (/5)** **‐Time‐varying** a	1.04 (1.01, 1.07)	0.007	1.23 (1.07, 1.42)	0.005
	**‐Baseline** c	1.20 (1.00, 1.42)	0.047		
	**MOCA** a (<22)	0.97 (0.65, 1.45)	0.89		
	**BDI** **‐Time‐varying** a **‐Baseline** c	1.33 (1.09, 1.62) 1.04 (1.01, 1.07)	0.005 0.005		
	**HADS‐ Anxiety** (≥8) **‐Time‐varying** a	2.05 (1.42, 2.95)	<0.001		
	**‐Baseline** c	1.93 (1.30, 2.86)	0.001		
	**Sniffin Score** a (Below 10^th^ centile)	1.49 (0.667, 3.32)	0.33		
	**QUIP** a ^**,**^ b	1.13 (0.81, 1.59)	0.48		
	**RBDSQ** a ^**,**^ b	1.20 (0.85, 1.67)	0.30		
	**ESS** a ^**,**^ b	0.83 (0.60, 1.16)	0.29		
	**Orthostatic Hypotension** a	0.98 (0.67, 1.42)	0.90		
	**Constipation** a	0.80 (0.58, 1.1)	0.17		
**Patient Factors**	**Age at symptom onset** (Years/3)^c^	0.87 (0.83, 0.92)	<0.001	0.89 (0.84, 0.94)	<0.001
	**Age at diagnosis** (Years/3)^c^	0.88 (0.83, 0.92)	<0.001		
	**Gender** c (Female)	1.61 (1.17, 2.22)	0.004	‐	‐
	**BMI** a *Continuous*	0.99 (0.96, 1.02)	0.51	0.95 (0.92, 0.99)	0.011
	<25	1.41 (1.02, 1.96)	0.037		
	**Smoking** c (Pack years prior to diagnosis)	1.00 (1, 1.01)	0.80		
	**Caffeine use** a **(**Cups per day)	1.00 (0.94, 1.07)	0.10		
	**QRISK2 vascular risk score** a ^**,**^ d (1‐4)	0.94 (0.75, 1.17)	0.58		
	**Socioeconomic status** a (5 point scale)	0.99 (0.86, 1.12)	0.82		
	**Education** a (>12 years)	1.04 (0.75, 1.43)	0.82		
	**No. of Cars** a (>2)	1.20 (0.87, 1.65)	0.27		
	**No. of Bedrooms** a (4+)	0.94 (0.68, 1.30)	0.70		
	**Job status** a (Supervisor)	0.74 (0.54, 1.03)	0.08		

Variables found to be significant (*P* < 0.05) on univariable analysis were tested in multivariable analysis. To aid in interpretation of exp(B), levodopa dose is expressed in increments of 100mg, MDS‐UPDRS scores in increments of 5, and age in increments of 3 years.

Exp(B); exponential beta. CI; Confidence Interval. LEDD; Levodopa Equivalent Daily Dosage. DA; Dopamine Agonist. MAOBI; Monoamine oxidase B Inhibitor. MDS‐UPDRS; Movement Disorders Society Unified Parkinson's Disease Rating Scale; MOCA, Montreal Cognitive Assessment; BDI; Beck's Depression Inventory. HADS, Hospital Anxiety and Depression Score; QUIP; Questionnaire for Impulsive‐Compulsive Behaviours in Parkinson's Disease. RBDSQ; REM Sleep Behaviour Disorder Screening Questionnaire. ESS; Epworth Sleepiness Scale. BMI; Body Mass Index.

a Time‐varying predictors.

b Once participants tested positive on QUIP, RBD questionnaire or ESS, they were considered positive in these tests for all subsequent time‐points.

c Time‐fixed predictors.

d Controlled for age.

**Table 3:** Predictors of motor fluctuations

	Variables	Univariable		Multivariable	
		Exp(B) (95% CI)	*P*	Exp(B) (95% CI)	*P*
**Time (Yrs)**	**Time**		<0.001		0.44
	<3.5	1			
	3.5‐5	1.33 (0.95, 1.86)	0.10	0.84 (0.52, 1.36)	0.48
	5‐6.5	2.72 (1.86, 3.99)	<0.001	1.06 (0.48, 2.34)	0.88
	≥6.5	1.65 (0.95, 2.86)	0.07	0.59 (0.19, 1.94)	0.39
**Medication Factors**	**Levodopa** **dose (mg/100)** a	1.25 (1.17, 1.34)	<0.001	1.22 (1.14, 1.32)	<0.001
	**Medication response** a (1‐Very much improved – 7‐very much worse)	0.53 (0.44, 0.63)	<0.001	0.54 (0.45, 0.66)	<0.001
	**Levodopa** **treatment** **duration (Years)** a	1.03 (0.95, 1.11)	0.45		
	**DA use** a	1.56 (1.16, 2.09)	0.003	‐	‐
	**MAOBI use** a	0.92 (0.67, 1.24)	0.57		
**Motor Features**	**MDS‐UPDRS II (/5)** ^**a**^	1.20 (1.09, 1.32)	<0.001		
	**MDS‐UPDRS III (/5)** ^**a**^	0.98 (0.93, 1.04)	0.47		
	**Disease Phenotype** a (Tremor Dominant)	0.89 (0.66, 1.20)	0.44		
**Non‐motor Features**	**MDS‐UPDRS I (/5)** **‐Time varying** a	1.14 (0.97, 1.35)	0.12	1.23 (1.03, 1.48)	0.024
	Interaction term with time^a^	1.19 (1.05, 1.36)	0.009	1.03 (1.00, 1.06)	0.041
	**‐Baseline** c	1.19 (1.02, 1.39 )	0.025		
	**MOCA** a (<22)	0.97 (0.65, 1.45)	0.89		
	**BDI** **‐Time‐varying** a **‐Baseline** c	1.03 (1.01, 1.05) 1.03 (1.01, 1.06)	0.002 0.006		
	**HADS‐ Anxiety** (≥8) **‐Time‐varying** a	2.19 (1.57, 3.05) 1.73 (1.21, 2.47)	<0.001 0.003		
	**‐Baseline** c	0.65 (0.37, 1.15)	0.14		
	**Sniffin Score** a (Below 10^th^ centile)	1.30 (0.96, 1.76)	0.09	‐	‐
	**QUIP** a ^**,**^ b	1.17 (0.87, 1.57)	0.31		
	**RBDSQ** a ^**,**^ b	1.37 (1.02, 1.83)	0.037	‐	‐
	**ESS** a ^**,**^ b	0.98 (0.70, 1.37)	0.889		
	**Orthostatic Hypotension** a	0.86 (0.65, 1.15)	0.305		
**Patient Factors**	**Age at symptom onset** (Years/3)^c^	0.88 (0.84, 0.92)	<0.001	0.89 (0.844, 0.938)	<0.001
	**Age at diagnosis** (Years/3)^c^	0.88 (0.84, 0.92)	<0.001		
	**Gender** c (Female)	1.34 (1.00, 1.80)	0.047	‐	‐
	**BMI** a *Continuous* <25	1.00 (0.97, 1.03)	0.93		
		1.24 (0.92, 1.67)	0.152		
	**Smoking** c (Pack years prior to diagnosis)	1.01 (1.00, 1.02)	0.32		
	**Caffeine use** a **(**Cups per day)	0.99 (0.93, 1.05)	0.66		
	**QRISK2 vascular risk score** a ^**,**^ d (1‐4)	1.08 (0.89, 1.32)	0.43		
	**Socioeconomic status** a (5 point scale)	1.40 (0.76, 2.58)	0.28		
	**Education** a (>12 years)	1.46 (1.09, 1.97)	0.012	1.72 (1.22, 2.41)	0.002
	**No. of Cars** a (>2)	1.32 (0.99, 1.76)	0.059	‐	‐
	**No. of Bedrooms** a (4+)	1.27 (0.95, 1.69)	0.11		
	**Job status** a (Supervisor)	0.80 (0.6, 1.08)	0.14		

Variables found to be significant (*P* < 0.05) on univariable analysis were tested in multivariable analysis. To aid in interpretation of exp(B), levodopa dose is expressed in increments of 100mg, MDS‐UPDRS scores in increments of 5, and age in increments of 3 years.

Exp(B); exponential beta. CI; Confidence Interval. LEDD; Levodopa Equivalent Daily Dosage. DA; Dopamine Agonist. MAOBI; Monoamine oxidase B Inhibitor. MDS‐UPDRS; Movement Disorders Society Unified Parkinson's Disease Rating Scale; MOCA, Montreal Cognitive Assessment; BDI; Beck's Depression Inventory. HADS, Hospital Anxiety and Depression Score; QUIP; Questionnaire for Impulsive‐Compulsive Behaviours in Parkinson's Disease. RBDSQ; REM Sleep Behaviour Disorder Screening Questionnaire. ESS; Epworth Sleepiness Scale. BMI; Body Mass Index; QRISK2, QRISK cardiovasular risk score version 2.

a Time‐varying predictors.

b Once participants tested positive on QUIP, RBD questionnaire or ESS, they were considered positive in these tests for all subsequent time‐points.

c Time‐fixed predictors.

d Controlled for age.

Higher levodopa dose, favorable medication response, younger age at symptom onset, and greater nonmotor symptom burden (as measured by MDS‐UPDRS I) were significantly associated with both dyskinesia and MF. Lower BMI was associated with dyskinesia, but not MF. Higher education level was associated with MF, but not dyskinesia.

Higher MDS‐UPDRS II was associated with dyskinesia and MF on multivariable regression. However, MDS‐UPDRS I was a stronger and more significant predictor than MDS‐UPDRS II, and the 2 scores were too closely correlated to be included in the same analysis.

The cumulative effect of increasing nonmotor severity (as measured by the MDS‐UPDRS I) was associated with MF risk and positively interacted with time, so this association increased further into the disease. Odds ratios for the association with time was 1.23 (95% confidence interval [CI], 1.04–1.49; *P* = 0.02), but there was evidence of a linear time interaction that was 1.03 (95% CI,1.00–1.06; *P* = 0.04) when both associations were included in a multivariable model, suggesting that the effect of the MDS‐UPDRS I becomes stronger over time.

Anxiety and low mood, measured by the Hospital Anxiety and Depression Scale–anxiety and Beck's Depression Inventory, respectively, were significant predictors on univariable analysis, but were not independent of one another. As MDS‐UPDRS I includes measures of mood and anxiety, these variables were not included in the multivariable analyses. The MDS‐UPDRS I, Beck's Depression Inventory, and Hospital Anxiety and Depression Scale–anxiety remained significant predictors of motor complications when their baseline values were included as time‐fixed variables.

### Dyskinesia Risk Calculator

A total of 62 participants were included in a validation study of the dyskinesia risk calculator designed by Schapira and colleagues.^5^ Of the 62 participants, 30 experienced dyskinesia within 200 weeks of commencing levodopa. Baseline demographics are described in Supporting Information Table S1. The episodic nature of the data collection meant that the MDS‐UPDRS II was measured up to 86 weeks before levodopa initiation (median 26 weeks). This interval was necessary to achieve a suitable sample size. The mean annual rate of change in MDS‐UPDRS II is 1.3 (SD = 2.9).

In the receiver operating curve analysis, the C‐statistic for dyskinesia risk score as a predictor of dyskinesia was 0.679 (95% CI, 0.545–0.813; *P* = 0.015). The C‐statistic is the area under the curve from plotting the true and positive rates against each other. A C‐statistic of 1 is a perfect model; 0.5 is equivalent to chance. The C‐statistic from the STRIDE‐PD study was slightly higher (0.697), reflecting that it was internally derived and hence likely to overestimate performance.

Two sensitivity analyses were performed to assess our selection method for bias. Using an interval of 150 to 190 weeks returned results comparable with 140 to 200 weeks (C‐statistic = 0.683; n = 55; 95% CI, 0.563–0.829; *P* = 0.021). Another analysis, including those who were only observed without dyskinesia before 140 weeks, also returned a comparable C‐statistic (0.685; n = 141; 95% CI, 0.585–0.785; *P* = 0.002).

## Discussion

### Incidence

Dyskinesia and MF are common even in this early PD cohort. Clinicians should consider the risk of motor complications from the outset of treatment. Other longitudinal studies generally report similar cumulative incidences at 5 years disease duration among levodopa‐treated participants.^10^ Variations may be explained by study design differences, such as the inclusion of participants not taking levodopa^15^ or the expression of time as a duration of treatment rather than disease.^9^, ^11^ These cohorts are significantly smaller than ours, with the largest including 189 patients at baseline.^10^

MF were more common than dyskinesia. This finding is consistent with most studies.^10^, ^16^ Our estimates of MF incidence are not grossly different from other cohorts.^10^, ^11^

MF are more pervasive and have greater functional impact than dyskinesia. It is the authors’ experience that many patients are unaware of mild dyskinesia, whereas MF and disease symptoms are more disabling. The evidence suggests that it is usually only severe, late‐stage motor complications that significantly affect quality of life.^3^

### Predictors

The predictors of motor complications can be divided into disease, medication, and patient factors.

#### Disease Factors

Our most interesting finding is the role of nonmotor features in predicting motor complication risk. Many studies, including ours, have demonstrated an association between motor disability, as measured by MDS‐UPDRS II, and motor complications.^6^, ^16^, ^17^ However, we know of only 1 small study to date that assessed the role of nonmotor features in predicting risk^18^ despite their significant contribution to disease burden. Our results suggest that overall nonmotor feature severity is highly correlated with motor complication risk and that the MDS‐UPDRS I is a predictor of future motor complication risk. Although it is difficult to compare motor and nonmotor scales directly, nonmotor symptom burden may be more clinically useful in predicting motor complication risk than motor symptoms. Nonmotor symptom scales often estimate a greater disease burden than motor scales.^19^ Because nonmotor symptoms are less responsive to levodopa,^20^ the MDS‐UPDRS I assessment of nonmotor aspects of daily living may be a more reproducible measure of disease severity among patients on treatment.

On assessment of individual nonmotor features, only anxiety and low mood were associated with motor complications, as noted elsewhere.^21^ Depression and anxiety carry similar factors as motor complications (eg, younger onset, motor severity).^22^ As anxiety in particular is a frequent wearing‐off symptom, this raises the possibility of reverse causation between anxiety and depressive nonmotor symptoms and motor fluctuations. To test this hypothesis, we looked at whether baseline MDS‐UPDRS I, Beck's Depression Inventory, and Hospital Anxiety and Depression Scale scores predicted future fluctuations, finding indeed a directional relationship between these variables and the future risk of motor complications. Characterizing the pathophysiology of this relationship is beyond the scope of this article, but these findings suggest the existence of a PD subtype at high risk of psychiatric disturbance and motor complications. Further studies are warranted to corroborate this finding and investigate this association.

A tremor‐dominant phenotype found by some,^23^ but not all,^15^ studies to be protective did not predict dyskinesia risk. Tremor‐dominant patients may take lower levodopa doses because of its poor antitremor effect.

#### Medication

As is widely accepted, levodopa dose was a strong predictor of dyskinesia.^1^ Perhaps counterintuitively, levodopa dose independently increased MF risk by a similar degree to dyskinesia, even though MF are thought to emerge as circulating levodopa reaches subtherapeutic levels.^24^ Trials that randomize patients to different levodopa doses have found the same effect,^16^, ^25^ suggesting a causative role for levodopa dose in MF risk.

Disease severity and levodopa dose are unsurprisingly correlated with both disease and treatment duration. In our model, the predictive value of disease or treatment duration was attenuated by severity and dose, suggesting that the increase in complication risk is the result not of time per se but of other time‐varying confounders such as dose and disease severity. Although it can be argued that time itself drives these variables in the absence of any disease‐modifying therapy. This contributes to the debate on whether levodopa therapy should be delayed and replaced by other (less effective and less well tolerated) medications such as DA, with the aim of postponing dyskinesia onset.^26^, ^27^ It is becoming increasingly recognized that any sustained reduction in risk by DA use can be explained by levodopa doses being lower because of DA supplementation at the expense of optimal symptom control.^28^, ^29^ Our study supports this by suggesting that dose and severity have greater predictive value than treatment duration.

Favorable levodopa response is associated with motor complications,^30^ suggesting a potential physiological link between levodopa response and adverse effects. Various clinical phenotypes exist within the PD spectrum, and both medication response and motor complication risk have been proposed as distinguishing features of such phenotypes.^31^ Pharmacogenetic variation throughout the levodopa pathway (eg, in the cathechol‐o‐methyltransferase [COMT] gene) has also been suggested to predict medication response and motor complication risk.^32^ Another contributing factor may be that greater benefit from treatment encourages concordance, increasing exposure to levodopa and its complications.

#### Patient Factors

Younger age at PD onset increased the risk of motor complications, irrespective of disease duration. This finding is consistent,^33^, ^34^ although the cause of this association is unclear.

Lower BMI is associated with an increased risk of dyskinesia,^16^, ^35^ but not MF.^16^ BMI was a time‐varying predictor, and this effect may be the result of both higher dose per kg of body weight, weight loss secondary to the hyperactivity of dyskinesia, or both as suggested in the literature.^35^, ^36^

Contrary to some studies^6^, ^10^, ^16^, ^34^ but consistent with others,^37^, ^38^ gender was not an independent predictor of either dyskinesia or MF. Caffeine intake, which other studies have shown to be protective,^11^, ^39^ had no effect in our study.

Higher education level was associated with increased MF risk. Until now, there has been little or no investigation of the impact of socioeconomic status and education on motor complication risk. We recognize, given multiple testing, the risk of type II error. Higher education levels may be associated with better treatment concordance, although this is debated.^40^ Further investigation is justified into the role of educational predictors of motor complications.

### Dyskinesia Risk Calculator

Our dataset provided an opportunity to validate the dyskinesia risk calculator designed by Schapira and colleagues^5^ using data from the STRIDE‐PD trial. Independent variables could not be recorded at the exact same time as levodopa commencement, and we recognize this could particularly limit the validity of the MDS‐UPDRS II measurement. However, given the low annual rate of change in MDS‐UPDRS II in our cohort, we feel the inclusion criteria for this analysis were similar enough to those of Schapira and colleagues^5^ to produce clinically relevant results.

There is a potential missing‐data problem in this analysis. Cases observed without dyskinesia before the window of 140 to 200 weeks, but not within it, were excluded because dyskinesia status after 140 weeks could not be determined. However, those observed with dyskinesia before 140 weeks were included regardless of follow‐up duration because, by the definition of survival analysis, they would remain dyskinetic thereafter. This should only create bias if the reason data being missing is related to dyskinesia onset. Clinically this is unlikely, and a second sensitivity analysis, including the excluded cases and assuming them to remain nondyskinetic, returns a very similar C‐statistic, so the risk of selection bias is low.

We found that the calculator is a valid tool but could be further improved. It is designed for use when commencing treatment and only estimates risk for the following 169 weeks. In our DTSA model, we found no association between gender and motor complications, and it may therefore not be necessary to include gender in the calculator if enough confounders are accounted for. Furthermore, we would speculate that age at commencing treatment is likely to only be an effective predictor as a surrogate for age at diagnosis (as younger onset PD participants are likely to also be younger at treatment initiation). Age at diagnosis may be a more relevant calculator variable. Finally, the calculator suggests that treatment duration is an independent predictor of dyskinesia. Our primary analysis suggests that this is unlikely to be the case. A more useful tool might include nonmotor predictors and medication response and be applicable to patients established on treatment.

### Strengths and Weaknesses

This study provides a robust longitudinal analysis model for predictors of motor complications in early PD. It is, to our knowledge, the largest of its type, allowing the assessment of a broad range of variables. Nevertheless, it has its limitations. DTSA models carry a risk of target events occurring in one interval but not being observed until a subsequent interval. This risk increases if participants are absent from a time interval because of delays in follow‐up. This occurred in 17 (9.14%) of 186 cases of dyskinesia and 24 (9.45%) of 254 cases of MF. The regression analyses remained valid.

Censoring is a limitation of any survival analysis. A proportion of the participants lost to follow‐up are likely to experience motor complications within 10 years of diagnosis. This limitation is acceptable if censoring is noninformative, that is, the onset of motor complications did not increase risk of loss to follow‐up. This assumption is reasonable as the functional impact of motor complications in early PD is minimal.^3^ Nevertheless, an element of informative censoring cannot be completely excluded.

The majority of covariates used are time varying rather than time fixed. In our opinion, this is a major strength of longitudinal models, as they provide information that remains clinically relevant throughout the duration of a chronic disease such as PD, informing treatment decisions based on the present clinical picture rather than historic data. Time‐fixed variables do carry a risk of reverse causality, and although this cannot be completely excluded, we have made efforts to demonstrate directionality for the variables at greatest risk of this (mood, anxiety, MDS‐UPDRS I).

## Conclusions

Dyskinesia and MF are common adverse effects of levodopa even in early PD. The predictors of motor complications can be divided into the following 3 categories: (1) disease factors, that is, severity of motor and nonmotor features (particularly mood and anxiety); (2) medication factors, that is, dose and clinical response; (3) patient factors, that is, age at onset, BMI (for dyskinesia), and education status (for MF). The duration of levodopa treatment itself does not increase the risk of dyskinesia. Alternative therapies may be considered in patients exhibiting these risk factors.

Attempts to create a calculator of dyskinesia risk appear valid but have room for improvement and require further validation and demonstration of utility in routine clinical care.

Further studies are warranted to evaluate the role of nonmotor disease factors and mood disturbance in predicting motor complications, the pathogenesis of MF, and the development of a robust risk calculator.

## Author Roles

1. Research Project: A. Conception, B. Organization, C. Execution; 2. Statistical Analysis: A. Design, B. Execution, C. Review and Critique; 3. Manuscript Preparation: A. Writing of the First Draft, B. Review and Critique.

M.K.: 1A, 1C, 2A, 2B, 3A

M.L.: 2A, 2B, 2C, 3B

F.B.: 1C, 3B

C.R.: 1C, 3B

T.B.: 1C, 3B

C.L.: 3B

J.K.: 1C, 3B

Y.B.S.: 1A, 1B, 2C, 3B

M.H.: 1A, 1B, 1C, 2C, 3B

## Full financial disclosures for the past 12 months

M.K. is employed at Oxford University Hospitals NHS Foundation Trust and in the past 12 months has been employed by Milton‐Keynes University Hospital NHS Foundation Trust. He is an honorary research fellow at the Oxford Parkinson's Disease Centre. He received an honorarium for a lecture delivered to the Parkinson Voice Project non‐profit organisation, Dallas, TX, on “Impulsivity and Creativity in Parkinson's.”

M.L. is employed at the University of Bristol and funded by grants awarded to the Oxford Parkinson's Disease Centre by Parkinson's UK. He received expenses reimbursement but no honoraria for an invited lecture at the Charcot Foundation conference. F.B. is employed at North Bristol NHS trust and in the past 12 months has been employed at Nottingham University Hospitals NHS Trust. He is a visiting academic fellow to the Oxford Parkinson's Disease Centre. C.R. is employed at Hampshire Hospitals NHS Trust. T.B. is a research fellow at the Oxford Parkinson's Disease Centre and receives funding from the Wellcome Trust Doctoral Training Fellowship. C.L. is a research fellow at the Oxford Parkinson's Disease Centre and receives funding from the NIHR Oxford Biomedical Research Centre. J.K. is employed at Oxford University Hospitals NHS Foundation Trust and receives funding from the NIHR Oxford Health Biomedical Research Centre. Y.B.S. is employed at the University of Bristol. He has received funding from the Gatsby Foundation, NIHR Health Technology Assessment Programme, Kidney Research UK, NIHR Health Technology Assessment Programme, and Parkinson's UK. He has received honoraria to speak at the Movement Disorders workshop. M.H. is employed at Oxford University Hospitals NHS Foundation Trust and is funded by Parkinson's UK and the NIHR Oxford Biomedical Research Centre. She is on the consultancy advisory board for Biogen and Roche Pharmaceutical companies.

## Supporting information


**Supplemental Table S1** Description of covariates and their collection methods.
**Supplemental Table S2.** Demographics of participants in the dyskinesia risk calculator validation study.

## Data Availability

Access to the data may be requested via the Oxford Parkinson's Disease Centre Data Access Committee. Initial inquiries can be made to the corresponding author.
