# Supplementary material for: Predictors of motor complications in early Parkinson's disease: A prospective cohort study
Source: Mov Disord. 2019 Jul 8;34(8):1174–83. doi: 10.1002/mds.27783 (PMC6771533; doi:10.1002/mds.27783)
Supplement: Supplementary file 1 — Supplemental Table S1 Description of covariates and their collection methods. Supplemental Table S2. Demographics of participants in the dyskinesia risk calculator validation study. [file MDS-34-1174-s001.docx]

| **Medication Factors** | | | |
| --- | --- | --- | --- |
| Levodopa dose | |  | Calculated using an established formula ^1^ |
| Treatment duration | |  | Time in years since first starting levodopa |
| **Motor features** | | | |
|  | | UPDRS III | 18 physician-reported questions on parkinsonian motor features seen on examination, each using a scale of 0-4 (absent, mild moderate, severe). Cumulative score was used as a representation of motor feature severity^2^. |
|  | | UPDRS II | 14 subject-reported questions on parkinsonian motor symptoms experienced in the past week, each using a scale of 0-4 (absent, mild moderate, severe). Cumulative score was used as a representation of motor feature severity^2^. |
| Motor phenotype | |  | Participants were classified as tremor-dominant or non-tremor dominant (NTD) based on UPDRS III score at each visit^3^. The NTD category included both postural instability and gait difficulty (PIGD) and indeterminate categories. |
| Response to medications | | Clinical Global Impression of Change | A scale of 1-7 grading a patient’s response to antiparkinson medication (Very much improved, much improved, minimally improved, no change, minimally worse, much worse, very much worse) ^4^. |
| **Non-motor features** | | | |
|  | | UPDRS I | 13 questions on common PD-related non-motor symptoms experienced in the past week, each using a scale of 0-4 (absent, mild moderate, severe). Cumulative score was used as a representation of non-motor feature severity^2^. |
| Cognition | | Montreal Cognitive assessment | Dichotomised into scores of <22 (possible dementia) and ≥22 ^5^ |
| Depression | | Beck Depression Inventory II (BDI) | Categorised into scores of 0-13 (minimal depression, 14-19 (mild depression), 20-28 (moderate depression) and >28 (severe depression) ^6^. |
| Anxiety | | Hospital Anxiety and Depression Scale (HADS) | Dichotomised into scores of ≥8 (probable anxiety) ^7^. In early visits, where HADS was unavailable, the Leeds anxiety and depression scale^8^ was used and scores were converted using equipercentile methods.^9^ |
| Sleep | | |  |
|  | Daytime somnolence | Epworth Sleepiness Scale (ESS) | Dichotomised where a score of ≥11 constitutes a positive result ^10^. Once participants scored positive, they were considered to be positive at all subsequent visits regardless of score. |
|  | REM sleep behaviour disorder (RBD) features | RBD Screening Questionnaire (RBDSQ) | Dichotomised where a score of ≥5 constitutes a positive result ^11^. Once participants scored positive, they were considered to be positive at all subsequent visits regardless of score. |
| Hyposmia | | Sniffin Sticks odour identification test | Dichotomised where scores below the 10^th^ centile constitute ‘hyposmia’ ^12^. |
| Autonomic | | Orthostatic hypotension assessment | Blood pressure measurements were taken after a patient lay flat for 3 minutes and after standing upright for 2 minutes. A systolic or diastolic drop of ≥20mmHg or ≥10mmHg respectively was considered positive ^13^. |
| Impulse control behaviours | | Questionnaire for Impulsive-Compulsive Behaviours in Parkinson’s Disease (QUIP) | The shortened QUIP-Anytime-PD-Short was used at first visit and QUIP-Current-PD-Short at subsequent visits^14^. A positive response in any category represented a positive result and once participants scored positive, they were considered to be positive at all subsequent visits regardless of subsequent responses. |
| Constipation | |  | Binary variable where having less than one bowel movement per day or requiring the use of laxatives in the previous week denoted constipation. |
| **Patient factors** | | | |
| Caffeine use, smoking | | Mini Environmental Risk Questionnaire for PD Baseline (MERQ-PD B) | Premorbid smoking history was recorded in pack years.  Current caffeine use was recorded in number of caffeine containing drinks per day ^15^. |
| Socio-economic status | |  | An ordinal scale from 0 to 4, where 4 represents more privileged socioeconomic status. One point for meeting each of the criteria; >12 years or higher level education, owning four or more bedrooms, owning two or more cars, working in a supervising job role. |

**Supplemental table 1.** Description of covariates and their collection methods.

1. Tomlinson, C. L. Stowe, R., Patel, S., Rick, C., Gray, R., Clarke C. E. Systematic review of levodopa dose equivalency reporting in Parkinson’s disease. Mov. Disord. 2010; 25: 2649–2653.
2. Goetz, C. G. et al. Movement Disorder Society-sponsored revision of the Unified Parkinson’s Disease Rating Scale (MDS-UPDRS): scale presentation and clinimetric testing results. Mov. Disord. 2008; 23: 2129–2170.
3. Stebbins, G. T. et al. How to identify tremor dominant and postural instability/gait difficulty groups with the movement disorder society unified Parkinson’s disease rating scale: Comparison with the unified Parkinson’s disease rating scale. Mov. Disord. 2013; 28: 668–670.
4. Guy, W. & National Institute of Mental Health (U.S.). Psychopharmacology Research Branch. Division of Extramural Research Programs. ECDEU assessment manual for psychopharmacology. (Rockville, Md. : U.S. Dept. of Health, Education, and Welfare, Public Health Service, Alcohol, Drug Abuse, and Mental Health Administration, National Institute of Mental Health, Psychopharmacology Research Branch, Division of Extramural Research Programs, 1976).
5. Hu, M. T. M. et al. Predictors of cognitive impairment in an early stage Parkinson’s disease cohort. Mov. Disord. 2014; 29: 351–359.
6. Beck, A. T., Ward, C. H., Mendelson, M., Mock, J. & Erbaugh, J. An inventory for measuring depression. Arch. Gen. Psychiatry. 1961; 4: 561–571.
7. Bjelland, I., Dahl A. A., Haug T. T., Neckelmann D., The validity of the Hospital Anxiety and Depression Scale: An updated literature review. J. Psychosom. Res. 52(2), 69-77 (2002).
8. Snaith, R., Bridge, G., & Hamilton, M. The Leeds Scales for the Self-Assessment of Anxiety and Depression. Br. J. Psychiatry. 1976; 128(2): 156-165.
9. Johns, M. W. A new method for measuring daytime sleepiness: the Epworth sleepiness scale. Sleep. 1991; 14: 540–545.
10. Nomura, T., Inoue, Y., Kagimura, T., Uemura, Y. & Nakashima, K. Utility of the REM sleep behavior disorder screening questionnaire (RBDSQ) in Parkinson’s disease patients. Sleep Med. 2011; 12: 711–713.
11. Hummel, T., Kobal, G., Gudziol, H. & Mackay-Sim, A. Normative data for the ‘Sniffin’ Sticks’ including tests of odor identification, odor discrimination, and olfactory thresholds: an upgrade based on a group of more than 3,000 subjects. Eur. Arch. Otorhinolaryngol. 2007; 264: 237–243.
12. Freeman, R. et al. Consensus statement on the definition of orthostatic hypotension, neurally mediated syncope and the postural tachycardia syndrome. Clin. Auton. Res. Off. J. Clin. Auton. Res. Soc. 2011; 21: 69–72.
13. Weintraub, D. et al. Validation of the questionnaire for impulsive-compulsive disorders in Parkinson’s disease. Mov. Disord. 2009; 24: 1461–1467.
14. Baig, F. et al. Personality and addictive behaviours in early Parkinson’s disease and REM sleep behaviour disorder. Parkinsonism Relat. Disord. 2017; 37: 72–78.

|  | **No Dyskinesia (n=32)** | **Dyskinesia (n=30)** |
| --- | --- | --- |
| **Gender (female, %)** | 7 (21.9%) | 13 (43.3%) |
| **Median age (years) (min – max)** | 67.9 (39.7 – 81.9) | 62.3 (49.1 – 77.9) |
| **Mean UPDRS II** **(min – max)** | 9.7 (2 – 21) | 10.0 (2 – 35) |
| **Median levodopa dose/weight (mg/kg)** **(min – max)** | 1.3 (0.49 – 3.26) | 1.7 (0.53 – 5.26) |
| **Median disease duration (Years)** **(min – max)** | 2.6 (1.6 – 5.0) | 2.7 (1.8 – 4.9) |
| **N on dopamine agonist (%)** | 16 (50.0%)^a^ | 14 (46.7%)^a^ |
| **N on MAOBI (%)** | 14 (43.8%)^b^ | 15 (50.0%)^b^ |
| **N on anticholinergic medications** | 0 | 0 |
| **N on COMTI** | 0^c^ | 2 (6.7%)^c^ |
| **Median dyskinesia risk score (min, max)** | **26.66(-0.47, 65.87)** | **33.89 (11.27, 74.86)** |

**Supplemental Table S2. Demographics of participants in the dyskinesia risk calculator validation study.**

^a,b,c^ p>0.05, Chi square analysis.
